# Mechanisms of Bone Resorption in Periodontitis

**DOI:** 10.1155/2015/615486

**Published:** 2015-05-03

**Authors:** Stefan A. Hienz, Sweta Paliwal, Saso Ivanovski

**Affiliations:** School of Dentistry and Oral Health, Griffith Health Institute, Griffith University, Gold Coast, QLD 4222, Australia

## Abstract

Alveolar bone loss is a hallmark of periodontitis progression and its prevention is a key clinical challenge in periodontal disease treatment. Bone destruction is mediated by the host immune and inflammatory response to the microbial challenge. However, the mechanisms by which the local immune response against periodontopathic bacteria disturbs the homeostatic balance of bone formation and resorption in favour of bone loss remain to be established. The osteoclast, the principal bone resorptive cell, differentiates from monocyte/macrophage precursors under the regulation of the critical cytokines macrophage colony-stimulating factor, RANK ligand, and osteoprotegerin. TNF-*α*, IL-1, and PGE_2_ also promote osteoclast activity, particularly in states of inflammatory osteolysis such as those found in periodontitis. The pathogenic processes of destructive inflammatory periodontal diseases are instigated by subgingival plaque microflora and factors such as lipopolysaccharides derived from specific pathogens. These are propagated by host inflammatory and immune cell influences, and the activation of T and B cells initiates the adaptive immune response via regulation of the Th1-Th2-Th17 regulatory axis. In summary, Th1-type T lymphocytes, B cell macrophages, and neutrophils promote bone loss through upregulated production of proinflammatory mediators and activation of the RANK-L expression pathways.

## 1. Introduction

Bone resorption is a basic physiologic process that is central to the understanding of many key pathologies, with its most common oral manifestation seen as the alveolar bone destruction in periodontitis [1–4]. This review aims to describe the prevailing understanding of mechanisms of bone resorption as related to periodontal disease, at the molecular and cellular levels. It outlines some of the newer advances in the field of osteoimmunology, and sheds light on recent research contributions and future directions from a clinical perspective [5–8]. Understanding the biological mechanisms that control the immunopathogenesis of the remodelling and resorptive processes will clarify not only the local control of bone cell function but also the pathophysiology of accelerated bone loss, as seen in periodontal disease and other immunoinflammatory diseases of bone such as osteoporosis and rheumatoid arthritis [9–11].

## 2. Bone Homeostasis and Maintenance

Bone is a remarkably dynamic and active tissue, undergoing constant renewal in response to mechanical, nutritional, and hormonal influences. A balance between the coupled processes of bone resorption by osteoclasts and bone formation by osteoblasts is required in a healthy adult [3, 12–14]. Under physiologic conditions, these processes are very carefully regulated by systemic hormones and local factors and orchestrated by osteocytes and bone lining cells which fine-tune interstitial fluid and plasma calcium levels [3]. Thus, bone resorption plays a major role in the homeostasis of skeletal and serum calcium levels, and the regulated coupling of resorption to new bone formation by osteoblasts is required for proper growth, remodelling, and skeletal maintenance [12–14]. The overall quality and quantity of bone will be affected by any factors that influence either of these processes or perturb this balance.

## 3. Bone Cells

Preosteoblasts, osteoblasts, osteocytes, and bone lining cells all arise from the osteogenic line of cells, which, in turn, arise from primitive mesenchymal cells in bone marrow stroma and from pericytes adjacent to connective tissue blood vessels. Their differentiation requires activation of the Osf2/Cbfa gene, which activates expression of osteocalcin, bone sialoprotein (BSP), osteopontin (OPN), and collagen synthesis, and is followed by stimulation from bone morphogenetic protein- (BMP-) 2 and transforming growth factor beta (TGF-*β*) [15–17]. Besides their primary role in bone formation, osteoblasts express chemokines, prostaglandins, and growth factors (e.g., BMPs, TGF-*β*, colony-stimulating factor- (CSF-) 1, granulocyte colony-stimulating factor (G-CSF), basic fibroblast growth factor (basic FGF), and insulin-like growth factor (IGF)) with autocrine, self-regulatory, and/or paracrine activity that regulate osteogenic as well as osteoclastic cells [18]. Osteoblastic cells have a major influence on the environmental responsiveness of osteoclasts through localisation, induction, stimulation, and inhibition of resorption [8, 13].

Osteocytes are mature bone cells that have become entrapped in bone matrix and mobilise calcium from matrix for transport and exchange with body fluids in response to systemic demand [19]. They too respond to systemic influences as evidenced by increased levels of cyclic AMP and act as transducers to modulate local bone remodelling activity [19]. They are liberated by osteoclasts during resorption, with the eventual fate of apoptotic cell death. Bone lining cells regulate the ionic composition of bone fluid, protect the bone surface from osteoclasts, and regulate new bone formation or resorption [12, 14].

Osteoclasts are highly specialised motile migratory bone resorptive cells, derived from haematopoietic stem cells [12–14, 20–22]. They are responsible for the degradation of mineralized bone and are, therefore, critical for normal skeletal growth and development, maintenance of bone integrity throughout life, calcium metabolism through remodelling, and homeostasis and repair [12, 14, 20]. As osteoclasts are the prime bone resorptive cells, local stimulation of their activity is an essential requirement for alveolar bone loss [23]. In response to key factors, such as M-CSF/CSF-1, osteoclast differentiation factor (ODF/RANKL), interleukins (IL), tumor necrosis factor (TNF), and contact with mineralized bone particles containing osteocalcin, haematopoietic precursors may undergo differentiation into monocyte and macrophage derived colony-forming cells, circulating peripheral blood monocytes and tissue macrophages, and finally fuse into mature multicellular osteoclasts [17, 20–22, 24–28].

One of the first events in the triggering of preosteoclasts is the contraction of the osteoblast actin and myosin cytoskeleton in response to local and systemic influences, for example, parathyroid hormone (PTH), retinoid acid, and vitamin D3 stimulation [8, 27]. This increases the width of intercellular spaces, exposing more osteoid to interstitial fluid. Osteoblasts also secrete collagenase and plasminogen activator [29]. IL-1, TNF, and epidermal growth factor (EGF) have been shown to deactivate osteoblasts and increase release of CSF-1 and RANKL [19, 27].

## 4. Cellular Mechanisms of Bone Remodelling: Resorption and Formation

The bone remodelling cycle operates continually as osteoclasts are constantly removing mature bone, with new bone simultaneously formed by osteoblasts [14]. This occurs throughout the skeleton in focal units called bone remodelling units (BRU), with each unit of activity lasting three to four months [9]. This multistep process functions in four distinct phases of activation, resorption, reversal, and formation.

“Activation” is the initiating event that converts a resting bone surface into a remodelling surface [12, 20]. It involves the recruitment of mononuclear osteoclast precursors to the bone surface and their differentiation and fusion into functional osteoclasts [8]. Terminal differentiation and mononuclear cell fusion is mediated by cell-to-cell interactions between osteoclast progenitors and osteoblasts/stromal cells and by contact with the mineral phase, particularly with osteocalcin [17, 27, 30]. Both CSF-1 and IL-1 stimulate preosteoclast fusion [26, 28]. E-cadherin is important for cell-to-cell adhesion associated with the fusion of preosteoclasts. Nonmineralised osteoid covering the mineralized bone matrix must be dissolved before the osteoclasts can attach to the mineralised matrix and initiate resorption [8]. Osteoblast proteases are responsible for dissolving this osteoid. Following this, the activated osteoclasts attach to the bone matrix and their cytoskeleton reorganizes; they take on a polarized morphology and form a sealing zone to isolate the resorption site and develop ruffled borders which secrete protease enzymes [9, 14, 20, 31].

During the “resorption” phase, osteoclasts work in concert removing both mineral and organic components of the bone matrix [14]. The hallmark of the resorbing surface is the appearance of scalloped erosion, called Howship's or resorption lacuna [12, 20]. The resorption phase lasts about 8–10 days, presumably the life span of the osteoclast [14].

Once most of the mineral and organic matrix has been resolved, there is a “reversal” phase lasting 7–14 days, marking the transition from destruction to repair. Here, the coupling of resorption to formation takes place [15]. After completion of one resorption lacuna, the osteoclast can move along the bone surface and restart resorption or undergo apoptosis [14].

Numerous paracrine and autocrine chemical signalling factors are involved in all aspects of remodelling, resorption, proliferation, and coupling. Coupling factors are released from their binding proteins during resorption by the acidic environment created by osteoclasts, and they further inhibit resorption via negative feedback, suppressing osteoclast formation and stimulating osteoblastogenesis [4, 19, 32]. Thus, in a series of locally controlled autoregulated cell activation events, a ten-day osteoclastic resorptive phase is usually followed by a repair phase of three months [19]. During repair, a cascade of differentiation events including chemotaxis, cell attachment, mitosis, and differentiation of osteoblast precursors takes place, leading to new bone deposition [19].

## 5. Bone Formation

Formation of new bone, a two-stage process, begins after a short reversal phase, commencing with the deposition of osteoid. The initial organic matrix consisting primarily (90%) of type 1 collagen and various other components is subsequently mineralised over a period of about 20 days [19]. Two theories generally elucidate how calcification proceeds: the matrix vesicle theory and the nucleation theory; it is speculated that both theories work in parallel depending on the type of skeletal tissue involved [3, 5, 12]. After the mineralisation process is triggered, the mineral content rapidly increases over the first few days to 75% of final mineral content, taking up to a year for the matrix to reach maximum mineral content. The primary constituent of the mature mineral phase is hydroxyapatite [9, 10, 19].

Noncollagenous bone matrix proteins play a key role in matrix mineralisation, cellular adhesion, and regulation of cell activity during coupling of formation and resorption. Osteocalcin, one of the most abundant of these proteins, has a vital role in mineralization, may act as a chemoattractant, and may be essential for osteoclast differentiation. Bone sialoprotein (BSP), a highly specific bone protein, has high calcium-binding potential, thus inhibiting mineral deposition. In addition, it promotes adhesion of osteoclasts to bone matrix molecules through the key RGD (arginine-glycine-aspartic acid) peptide sequence and may regulate osteoclast formation. Osteopontin and osteonectin too are important in osteogenic cell activity [15–17, 19].

## 6. Degradation of the Mineral and Organic Matrix

Osteoclasts resorb bone in resorption lacunae by generating a pH gradient between the cell and bone surface, favouring the mineral-dissolving action of the osteoclast proteinases. Carbonic anhydrase (CA) II is the main cytoplasmic source of protons for the acidification of the lacuna. This hydrates carbon dioxide to carbonic acid, which ionizes into carbonate and hydrogen ions [9, 10, 12, 14, 19, 22]. A vacuolar-type proton pump, V-ATPase, transports the protons generated by CAII into intracellular vesicles. These are then transported and fused to the RB membrane, releasing their proton content to the lacuna. Acidification is subsequently completed by passive potential driven chloride transport. The chloride channel of the ruffled border is identified as ClC-7, and it is transported along with the proton pump to the RB via endosomes [5, 9, 19, 33, 34].

The basolateral membrane exchangers Na^+^ and Cl^−^  HCO_3_^+^ maintain internal pH at physiologic levels. Calmodulin, a cytoplasmic calcium-binding protein concentrated in the osteoclast cytoplasm adjacent to the RB, regulates the effects of intracellular calcium and the ATP-dependent proton transport across the RB. As resorption proceeds, the increase in cytoplasmic calcium ultimately deactivates the osteoclast, triggering cell detachment from the bone matrix and loss of the RB [9, 19, 33, 34]. Solubilization of hydroxyapatite is followed by digestion of the exposed organic matrix by lysosomal enzymes, bone-derived collagenases, and proteinases [14, 29]. Osteoclasts contain the highest concentration of mitochondria of any cell type, thus generating the ATP required for the carbonic anhydrase-catalysed production of hydrogen ions [9].

Degradation products are removed by transcytosis and finally released into the extracellular space. The specific enzyme TRAP (tartrate-resistant acid phosphatase) is located in cytoplasmic vesicles, which fuse to the transcytotic vesicles to destroy the endocytosed material. When the osteoclast moves away from the resorption lacuna, phagocytes clean up the debri, and osteoblasts move in to begin bone formation anew [19]. The dissolution of the mineral phase in the acidic microenvironment below the RB exposes collagen fibrils to the enzymatic attack of cathepsins B, E, K, S, and L. These cysteine proteases are secreted by osteoclasts to degrade native collagen at an acidic pH of 4.5 [19]. Thereafter, matrix metalloproteinases (MMPs), such as gelatinase A (MMP-2), stromelysin (MMP-3), and collagenase (MMP-1), continue with the matrix degradation process. Thus, calmodulin antagonists and MMP inhibitors can block resorption by inhibiting acidification of the resorptive compartment [19, 29].

## 7. Regulation of Osteoclastic Bone Resorption

The rate of bone resorption can be regulated either at the level of differentiation of osteoclasts from their hematopoietic precursor pool or through the regulation of key functional proteins which control the attachment, migration, and resorptive activities of the mature cell [8, 10, 12, 22]. It is becoming evident that many of the cellular events involved in resorption of bone are modulated by a group of local osteotropic factors which have extremely potent effects on bone cells both* in vitro* and* in vivo*. It must be recognized that many of these cytokines and growth factors exhibit significant redundancy and pleiotropy or overlap in their local effects [18, 19, 35].

As emphasised previously, in states of disease, a disturbance in the homeostatic balance that is essential for functional bone turnover results in destructive osteolytic processes. In inflammatory periodontal disease, both microbial and host-derived factors are implicated in the bone resorption and remodelling processes [2, 4, 8]. These chemical modulators play highly complex roles, and several cell types are often involved. It is difficult to definitively ascertain the precise role of a cytokine or growth factor* in vitro*, and even less so* in vivo*, as multiple local factors often modify the* in vivo* effect [7, 20]. Some factors act directly on osteoclastic cells, whereas others act indirectly through other cell types in the local environment or through secondary production of additional factors [18].

Cytokine regulation is likely to be more important for trabecular bone, which is closer to the cytokine-rich marrow than cortical bone [3, 19]. Many potent osteotropic cytokines, such as IL-1, IL-6, TNF-*α*, and TGF-*β*, mediate a multitude of effects in the body in addition to their effects on bone cells [19]. The production of cytokines by osteoblasts is regulated by various hormones and cytokines along with bacteria and lipopolysaccharide [19, 28]. These too can act synergistically with local factors to influence the bone homeostatic balance [20]. Thus, numerous hormones, growth factors, and cytokines modulate osteoclast activity by regulating their differentiation, activation, life span, and function. These include parathyroid hormone (PTH), calcitriol, PTH-related protein, PGE_2_, thyroxine, and IL-11 [3, 19, 36].

The proinflammatory cytokines (IL-1 and IL-6, TNFs) have been implicated in the stimulation of osteoclastic resorption in periodontitis. The functions of the immunoregulatory cytokines (IL-2 and IL-4, interferon gamma) are less clear, but low levels of these may contribute to periodontitis. Genetic factors have been shown to account for up to 80% of control of bone mineral density, thus playing a major role in determining variation. However, it is the rate of bone formation rather than the rate of resorption that is influenced by genes [2, 4, 19]. Some individuals demonstrate aggressive bone destruction and high levels of proinflammatory and bone resorptive cytokines that cannot be completely explained by presence of pathogenic bacteria alone. Genetic variation, termed single-nucleotide polymorphisms, of key immune or inflammatory regulatory factors may explain these variances in periodontal disease manifestation, as well as the familial aggregation of aggressive forms of the disease [19].

Systemic influences on bone resorption may be exerted by several mediators, including PTH, IL-1, TNF, TGF, and 1,25-dihydroxyvitamin D_3_. These factors may affect osteoclast number and activity, directly influence osteogenic cells to cause cytoplasmic contraction and secretion of collagenase, tissue plasminogen activator and RANK-L. Of note, the C-terminal fraction of PTH has been shown to increase osteoclast formation and activity in the presence of osteoblasts and accelerate osteoclast-like cell formation from hematopoietic precursors in the absence of osteoblasts. Calcitonin, interferon gamma (IFN *γ*), and TGF *β* are potent inhibitors of osteoclast activity and differentiation [27, 29, 36–38]. The hormones PTH and calcitonin act in concert to maintain blood calcium concentrations at normal physiological levels (0.5–10.5 mg/dL), with actions on intestinal absorption and renal excretion as well as bone cells. There is evidence to support a direct effect of PTH on osteoclasts; however, there is much evidence that supports an indirect mechanism, whereby PTH stimulates osteoblasts to release RANKL, which subsequently activates osteoclasts. PTH also stimulates osteoblastic production of IL-6, which increases osteoclastic differentiation, and causes osteoblasts to contract making the bone surface more susceptible to resorption [6, 19, 25, 34, 36].

The polypeptide calcitonin increases cellular calcium and cAMP and disrupts the clear zone cytoskeleton by decreasing the size of the RB and altering podosome binding ability. It blocks proton extrusion and decreases osteopontin expression; hence osteoclasts are seen to detach from bone surfaces within 15 minutes of its administration. The sex steroids exert an anabolic effect by stimulating osteoblast proliferation and differentiation, as well as decreasing IL-6 transcription. Postmenopausal women experience osteoporosis due to increased osteoclastic resorption and decreased osteoblast proliferation [9, 19, 32, 33].

## 8. Local Mediators of Bone Resorption

Local formation of osteoclasts and their stimulation are required for alveolar bone loss. It has been shown that multiple mediators, such as IL-1, IL-6, IL-11, IL-17, TNF-*α*, TNF-beta, TGF-*β*, kinins, and thrombin, can stimulate bone resorption [4, 7, 13, 23]. Bone resorption is also directly regulated locally by ionized calcium generated as a result of osteoclastic resorption, and new evidence indicates that endothelial cells may also play a part via mediators including nitric oxide and endothelin [33]. Lipid mediators, such as bacterial lipopolysaccharide, host-derived platelet-activating factor, and prostaglandins, may also be involved in stimulation of bone resorption. Reactive oxygen intermediates and extracellular nucleotides, both present at sites of inflammation, have also been implicated [8].

The roles of local inflammatory mediators generated by macrophages and T lymphocytes in bone resorption have been extensively studied. Their effector functions on tissue can be direct or indirect as recently reported in relation to the osteoblast stimulated RANKL-production pathway. Alveolar bone resorption in periodontitis can thus be directly or indirectly induced by the cellular inflammatory infiltrate [4, 11, 23, 24].

The gingival crevicular fluid (GCF) has been shown to contain a complex array of protein components that not only irrigate the gingival sulcus but also are released into the oral cavity [39, 40]. GCF is derived from gingival capillary beds (serum components) and from both resident and emigrating inflammatory cells. This fluid contains an array of innate, inflammatory, and adaptive immune molecules and cells whose role is to contribute to the interaction of host and bacteria in this ecological niche [40]. Studies demonstrate that GCF contains mediators that can stimulate bone resorption* in vitro*. The primary factor responsible appears to be IL-1*α*, with IL-1*β* and PGE_2_ also significant. This exudate, with diagnostic and prognostic potential, is an accessible source of extracellular matrix derived biologic markers of periodontal bone resorption [18, 24, 41–43].

Analysis of GCF has identified cell and humoral responses in both healthy individuals and those with periodontal disease. Although there is no direct evidence of a relationship between GCF cytokine levels and disease, interleukin-1 alpha (IL-1*α*) and IL-1*β* are known to increase the binding of PMNs and monocytes/macrophages to endothelial cells, stimulate the production of PGE_2_ and the release of lysosomal enzymes, and stimulate bone resorption [42]. Preliminary evidence also indicates the presence of interferon-*α* in GCF, which may have a protective role in periodontal disease because of its ability to inhibit the bone resorption activity of IL-1*β* [44, 45]. Pyridinoline cross-links, in particular, are specific for bone resorption and thus useful in differentiating gingival inflammation from bone destruction in active lesions [18].

## 9. Roles of Receptor Activator of Nuclear Factor-*κ*B Ligand (RANKL) and OPG

The activation and differentiation of osteoclasts are modulated by three members of the TNF ligand and receptor superfamilies: the osteoclastogenesis inducers RANKL, RANK, and OPG. Identification of these three peptides has contributed enormously to our understanding of the molecular mechanisms of osteoclast differentiation and activity [6, 11, 22, 24, 25, 35, 46–48]. RANKL (receptor activator of nF-*κ*B ligand) is a member of the TNF superfamily (also known as osteoclast differentiation factor: ODF, TRANCE, and TNFSF-11). It is expressed as a membrane bound protein (mRANKL) or in soluble form (sRANKL) by osteoblasts or stromal cells. When RANKL binds to its receptor, RANK, on osteoclast and preosteoclast cell surfaces, it promotes osteoclast formation by stimulating proliferation and differentiation [9, 22, 25, 27, 47].

Osteoprotegerin (OPG), its decoy receptor, is a circulating protein, produced by a variety of cell types including osteoblasts and marrow stromal cells, which inhibits osteoclast formation by binding mRANKL, thereby preventing the stimulatory cell-to-cell interaction with preosteoclasts and inhibiting RANKL/RANK interactions. Hence, these three proteins are essential for osteoclast differentiation directed by osteoblasts, and the balance between RANKL and OPG in osteoblasts directs new osteoclast recruitment [7, 25, 32, 46, 47].

Other resident periodontal cells including ligament and gingival fibroblasts also participate in the regulation of bone remodelling and resorption. Infiltrated leukocytes produce inflammatory mediators, for example, IL-1 and PGE_2_, which affect RANKL and OPG expression by osteoblasts, periodontal ligament fibroblasts, and gingival fibroblasts. RANKL is also expressed in activated T cells [11, 24, 25, 46, 48, 49].

A key finding of recent studies is that sRANKL in combination with CSF-1/M-CSF stimulates osteoclast development from peripheral blood cell precursors by binding to its receptor. It has been established that osteoblasts are responsible for producing CSF-1 and that contact between osteoblasts and osteoclast precursors, mediated by critical survival factor CSF-1 and its receptor, promotes osteoclast development [5, 26–28, 38, 49].

Activation of the RANKL receptor increases the expression of TRAP, *β*_3_ integrins, cathepsin K, and calcitonin receptors on preosteoclasts. Thus, OPG is a negative regulator and RANKL a positive regulator of osteoclastogenesis through interaction with appropriate receptors on cells of the monocyte and macrophage cell lineage. In addition, many of the local and systemic regulators of osteoclastic resorption have been shown to act via the RANKL/OPG and CSF-1 pathways [24, 27, 46, 47].

## 10. Immunopathogenesis of Periodontal Disease

In chronic periodontal disease, biologically active substances within bacterial plaque induce a local inflammatory response in the gingival soft tissues and periodontium [2]. The resultant influx of inflammatory cells produces a host of cytokines, for example, PGE_2_, IL-1, and RANK-L, that promote resorption through osteoclasts, the primary bone resorbing cell. Thus, in pathologic inflammatory conditions, stimulatory inflammatory cell products initiate osteoclast activity and disturb the fine balance between protective and destructive processes [3, 6, 14, 22, 25, 36]. This is termed the immunopathogenesis of periodontal disease, and pioneering research in this area over the last decade has spawned a whole new field termed “osteoimmunology” [37, 50].

In periodontal disease, the cellular inflammatory infiltrate of T cells, B cells, macrophages, and neutrophils within gingival connective tissue is increased, with a concurrent increase in the secretion of inflammatory mediators [1, 51]. These inflammatory cells also interact with stromal cells, such as osteoblasts, periodontal ligament, and gingival fibroblasts. RANKL-mediated osteoclastogenesis plays a pivotal role in inflammatory bone resorption, and its expression is increased in periodontitis [22, 24]. While lymphocytes produce RANKL, they might not be involved in bone resorption under physiological conditions. However, in inflammatory pathological resorptive states, activated T lymphocytes may mediate bone resorption through excessive production of sRANKL, and findings suggest that activated T and B lymphocytes are one of the major RANKL-expressing sources in diseased periodontal tissue [11, 22, 25, 27]. Numerous animal models support this association. The majority of RANKL produced by T cells may be soluble, as the expression of mRANKL on T cells is limited. Another key finding is an increase in osteoclast numbers on the alveolar bone crest of animals receiving antigen-specific lymphocytes, which can be suppressed by OPG [11, 47, 48].

Gingival fibroblasts are heterogenic in that they produce OPG in response to LPS and IL-1, suggesting a protective role to suppress osteoclast formation; however, they may also augment chronic inflammatory processes through IL-6 and IFN production. The periodontopathic bacteria* Aggregatibacter actinomycetemcomitans* (*Aa*) and* Porphyromonas gingivalis* (*Pg*) have unique mechanisms to induce RANKL in osteoblasts and gingival fibroblasts. When stimulated with LPS and IL-1, osteoblasts and periodontal ligament fibroblasts express RANKL. RANKL and OPG expression may also be related to the function of amelogenin and regulation of odontoclast formation [11, 25, 28, 30]. Recently, it has been shown that RANKL is upregulated whereas OPG is downregulated in periodontitis compared to periodontal health, resulting in an increased RANKL/OPG ratio. This ratio is further upregulated in smokers and diabetics [52]. It has also been reported that the molecular mechanisms of T cell mediated regulation of osteoclast formation occurs through cross-talk signalling between RANKL and IFN-*γ*. Indeed, IFN-*γ* produced by T cells induces rapid degradation of the RANK adapted protein, TNF receptor associated factor 6 (TRAF6), which results in strong inhibition of RANKL induced activation of the transcription factor NF-*κ*B and c-Jun N-terminal kinase [9].

## 11. Role of Specific Immune Cells 

CD4+ and CD8+ T cells are present in periodontal lesions, as are memory and activated T lymphocytes, and different T cell subsets appear involved in either up- or downregulation of RANKL-mediated periodontal bone resorption. Moreover, Th1- and Th2-type T lymphocytes and their associated cytokines may be present, with a polarization towards a Th1 profile [9, 48, 49, 53]. It has been proposed that Th1-type cells promote bone loss, as RANK-L appears to be predominantly expressed on Th1-type cells, while regulatory T cells suppress T helper type 1 mediated bone loss. The production of proinflammatory cytokines IL-1 and TNF-*α* is upregulated by Th1-type T cells. These can induce bone resorption indirectly by stimulation of osteoclast precursors and subsequent activation of osteoclasts through RANK-L production by osteoblasts [49, 53]. Activated T cells can also, through production and expression of OPG, directly promote osteoclast differentiation. These direct and indirect modes of T cell involvement in periodontal bone resorption appear dependent on the extent of Th1-type T cell recruitment in inflamed tissues [53]. It has been well recognized that control of this shift is mediated by a balance between the so-called Th1 and Th2 subsets of T cells, with chronic periodontitis being mediated by Th2 cells [50]. More recently T regulatory (Treg) and Th17 cells have been demonstrated in periodontal tissues, suggesting a role for these mediators in the immunoregulation of the disease [1, 50]. However, Th17 and IL-17 have also been shown to display a protective role as well as a destructive role in periodontal bone resorption [54, 55]. Different models of inflammation report opposite functional roles of IL-17 in terms of its effects on bone destruction. In a recent study it was concluded that IL-17 is protective in the development of periapical lesions depending on its regulation of myeloid cell mediated inflammation. However, the authors noted that the detailed mechanism behind the IL-17 signal-mediated protection in periapical lesions remains unclear [54].

## 12. Bacterial Influence

Similar to other polymicrobial diseases, periodontitis is now characterized as a microbial-shift disease owing to a well-characterized change in the microorganisms that are present (from mostly Gram-positive to mostly Gram-negative species) during the transition from periodontal health to periodontal disease [56, 57].

In a milestone study by Socransky et al. using whole-genome DNA probes, several bacterial complexes associated with either periodontal health or disease were identified. This included three bacterial species that were designated, the “red-complex” periopathogens—*Porphyromonas gingivalis*,* Tannerella forsythia*, and* Treponema denticola*—which grouped together in diseased sites and showed a strong association with disease [58]. Much research has been directed towards understanding the pathogenic mechanisms and virulence determinants of these three bacterial species in the context of a conventional host-pathogen interaction, as exemplified by diseases with single-infective agent aetiology [59]. Support for the alternative hypothesis that periodontal pathogens transform the normally symbiotic microbiota into a dysbiotic state, which leads to a breakdown in the normal homeostatic relationship with the host, comes from evidence that* P. gingivalis* has evolved sophisticated strategies to evade or subvert components of the host immune system (e.g., Toll-like receptors (TLRs) and complement), rather than acting directly as a proinflammatory bacterium [60]. In other words,* P. gingivalis* could be a keystone pathogen of the disease-provoking periodontal microbiota [61–63].

The pathogenic processes of periodontal diseases are primarily due to the host response, which propagates the destruction initiated by microbes. Harmful pathogenic products and enzymes such as hyaluronidases, collagenases, and proteases break down extracellular matrix components in order to produce nutrients for their growth [2, 6, 11]. Arg- and Lys-gingipain cysteine proteinases produced by* P. gingivalis* are key virulence factors that lead to host tissue invasion. Once immunoinflammatory processes begin, various molecules (e.g., proteases, MMPs, cytokines, prostaglandins, and host enzymes) are released from leukocytes and fibroblasts. An imbalance between the level of activated tissue-destroying MMPs and their endogenous inhibitors (TIMPs) has been demonstrated. Thus, the connective tissue attachment and alveolar bone are destroyed, and the junctional epithelium and the inflammatory infiltrate migrate apically [7, 34, 40, 64, 65]. In addition, osteoclasts are activated, initiating bone destruction through direct mechanisms and indirectly through RANKL, RANK, and OPG modulation. In the presence of periodontopathogens, CD4+ T cells show increased RANKL expression [11, 24, 25, 46, 49]. As the destructive pattern continues, subsequent increase in microbial density propagates the periodontal lesion. The flora progressively becomes more anaerobic, and the host response becomes more destructive and chronic. Eventually bone loss and the destructive lesion progress to an extent that can lead to tooth loss [2, 7].

Bacterial virulence factors are capable of potentiating bone resorption themselves. Endotoxin from Gram-negative cell walls activates CD4+ T cells to stimulate resorption via their interaction with macrophages.* Pg* produces a fimbrial protein that is a potent osteoclast stimulator via a tyrosine kinase mechanism [8], antibodies against which prevented bone loss in infected animals.* Aa* produces a 62 kDa heat shock protein associated with the ability to stimulate bone resorption at picomolar concentrations, as well as a peptide that acts as a potent IL-6 inducer in fibroblasts and monocytes [7, 9, 10]. Other virulence factors of* Treponema denticola* and* T. forsythia* include the binding of FH, a negative regulator of complement, to spirochetal surface proteins, that correlates with complement resistance [66–68]. Recently,* T.denticola* has been shown to produce cystalysin, an enzyme that catalyzes the *α*,*β* elimination of L-cysteine to produce pyruvate, ammonia, and sulfide, which in turn enables the bacterium to produce sulfide at millimolar concentrations in the periodontal pocket. Sulfide is responsible for hemolytic and hemoxidative activities and for the damage to the gingival and periodontal tissues. Moreover, sulfide creates an ecological niche that selectively benefits* T.denticola* [69].

*T. forsythia* expresses a uniquely glycosylated surface envelope, known as the surface- (S-) layer, which plays an immunomodulatory role in influencing the immune response [70]. This S-layer has recently been shown to be important in delaying the cytokine responses of monocyte and macrophage cells* in vitro* [71, 72]. Settem et al. demonstrated in a mouse model of periodontitis that a terminal pseudaminic acid and N-acetylmannosaminuronic acid containing trisaccharide branch on an O-glycan core linked to the* Tannerella* surface proteins plays a role in dampening Th17 differentiation and mitigating neutrophil infiltration into the gingival tissue [73].

## 13. The Innate Immune Response, TLRs, and PAMPs

The host response against periodontopathic bacteria consists of innate and acquired immunity. The innate response meets the challenge of discriminating among large numbers of pathogens through recognition of conserved evolutionary molecular motifs called PAMPs (pathogen associated molecular patterns), which are expressed on pathogens but not by the host. The recently discovered Toll-like receptors (TLRs) are pattern-recognition receptors with key roles in detecting microbes and initiating inflammatory and host defense responses [74]. These signalling receptors are critical in pathogen recognition by the host, through specificity of recognition for several important PAMPs [7, 57]. TLRs are expressed by myelomonocytic cells, endothelial cells, epithelial cells, and other cells, including gingival fibroblasts [7]. Examples of PAMPs that are recognized by TLRs include peptidoglycan bacterial lipoproteins and* Pg* LPS (TLR-2), double stranded ribonucleic acid, LPS and heat shock proteins (TLR-4), and flagellin (TLR-5) [57, 74].

The TLR-PAMP interaction results in the recruitment of specific adapter molecules, which then bind the IL-1R-associated kinase. The signal is transmitted through a chain of signalling molecules common to all TLRs, involving tumor necrosis factor receptor-associated factor-6 (TRAF6) and mitogen activated protein kinases (MAPKs) [30, 74]. Subsequent activation of RANK and activated protein-1 leads to transcription of genes involved in stimulating the innate defenses, for example, expression of intercellular adhesion molecule-1 (ICAM-1) and lymphocyte function associated antigen-1 (LFA-1), causing greater attachment and migration of leukocytes to tissues, as well as increased expression of proinflammatory cytokines involved in significant downstream bone resorption [30, 53]. IL-8 expression attracts neutrophils, and activation of endothelial cells, macrophages, dendritic cells, and neutrophils stimulates matrix metalloproteinase production, with a direct mechanism of tissue damage. Macrophages too when stimulated by pathogenic peptides are directly activated, producing various cytokines and biological mediators, for example, MMP-1 and nitric oxide [7, 11, 75].

Collective data over the last few years provide evidence that gingival fibroblasts and periodontal ligament cells are equipped to respond to LPS stimulation through TLR-PAMP recognition and involvement of the RANKL-mediated responses, producing various inflammatory cytokines, such as IL-1, IL-6, and IL-8, when stimulated by oral bacterial LPS fractions from pathogens such as* Pg*,* Pi* (*Prevotella intermedia*), or* Aa* [7, 11, 74–76].

Osteoblasts, which are highly sensitive to PAMPS, can also be induced to produce mediators and cytokines that are involved in bone resorption, as well as inhibition of the protective factor OPG.* Pi* LPS inhibits differentiation of osteoblasts and mineralisation of bone. Cementoblasts, however, when stimulated by LPS, exhibit decreased levels of RANKL and increased expression of both OPG and osteopontin, suggesting a protective mechanism against bone and root resorption [7, 30, 40].

In addition, LPS from different periodontopathogens, CPg DNA, and* Aa* capsular polysaccharide promote osteoclast differentiation from bone marrow cells. Monocytes stimulated by PAMPS demonstrate increased differentiation into osteoclasts, and induced RANKL expression plays a central role [30, 74]. Costimulatory factors, for example, GM-CSF and M-CSF, are also important as is the secretion of IL-12, which activates T cells to produce IFN gamma, leading to development of the cell mediated Th1 T cell response. In contrast, without costimulatory factors, the Th2 response predominates [28, 49, 50]. Recently, the effects of* P. gingivalis* LPS1435/1449 and LPS1690 on the expression of TLR2 and TLR4 signal transduction and the activation of proinflammatory cytokines IL-6 and IL-8 in human gingival fibroblasts were investigated and it was suggested that these lipid* Aa* structures differentially activate the TLR4-mediated NF-*κ*B signaling pathway and significantly modulate the expression of IL-6 and IL-8 [77]. A study aimed at identifying ligands on the surfaces of intact* P. gingivalis* cells that determine their ability to activate TLR2 found that it is due to a lipoprotein contaminant [78]. Further, a number of reports have proposed that the expression of an antagonistic or immunologically inert lipid A by* P. gingivalis* is a mechanism for evasion of TLR4 signaling [79].

Thus, in summary, LPS induced disease leads to the initiation of a local host response in gingival tissues that involves recruitment of inflammatory cells, generation of prostanoids and cytokines, elaboration of lytic enzymes, and activation of osteoclasts. Specifically, LPS increases osteoblastic expression of RANKL, IL-1, PGE_2_, and TNF-*α*, each of which is known to induce osteoclastic activity, viability, and differentiation [11, 28, 30, 50, 75, 76]. A variety of immune associated cell populations are responsible for the pathogenic processes in periodontal tissues, including specific CD4+ T cells, recruited monocytes, macrophages, and fibroblasts. These produce cytokines (TNF-*α*, IL-1 *β*, etc.) within the lesion, which can be monitored and detected in the circulating GCF. In turn, these cytokines are pivotal to the destructive cascade and ultimately trigger the production of MMPs, prostaglandins, and osteoclasts. The end result is irreversible damage to the tooth supporting soft tissues and alveolar bone [23, 40, 43].

## 14. Conclusion

Bone resorption via osteoclasts and bone formation via osteoblasts are coupled, and their dysregulation is associated with numerous diseases of the skeletal system [3, 4, 13]. A wide range of host and microbial factors contribute to alveolar bone loss in periodontitis [2, 4, 23]. Yet, much remains to be understood about the complex mechanisms whereby these factors regulate bone resorption in periodontitis [7, 75]. Recent developments in the area of biological processes and mediators of osteoclast differentiation and activity have expanded our knowledge of resorption processes and set the stage for new diagnostic and therapeutic modalities to treat situations of localized bone loss as seen in periodontal disease [7, 25, 52, 62, 75, 80, 81].
